# Introducing the concept of a new pre-referral treatment for severely ill febrile children at community level: a sociological approach in Guinea-Bissau

**DOI:** 10.1186/1475-2875-13-50

**Published:** 2014-02-06

**Authors:** Audrey Vermeersch, Anaëlle Libaud-Moal, Amabelia Rodrigues, Nicholas J White, Piero Olliaro, Melba Gomes, Elizabeth A Ashley, Pascal Millet

**Affiliations:** 1 EA 4575 Développements Analytiques et Pharmaceutiques appliqués aux Maladies Négligées et aux Contrefaçons, Université Bordeaux Segalen, Bordeaux, France; 2 Projecto de Saúde de Bandim, Bissau, Guinea-Bissau; 3 Faculty of Tropical Medicine, Mahidol University, 420/6 Rajvithi Road, Bangkok 10400, Thailand; 4 Centre for Vaccinology and Tropical Medicine, Churchill Hospital, Oxford OX3 7LJ, UK; 5 WHO Special Programme for Research and Training in Tropical Diseases (TDR), United Nations Development Programme (UNDP)/World Bank, Geneva, Switzerland

**Keywords:** Severe febrile illnesses, Malaria, Artesunate, Antibiotics, Rectal administration, Pre-referral treatment, Rural areas

## Abstract

**Background:**

Innovative strategies are needed to tackle childhood mortality in the rural tropics. Artesunate suppositories were developed to bring emergency treatment closer to severely ill children with malaria in rural areas where injectable treatment is not possible for several hours. Adding an antibacterial rectal drug would extend this strategy to treat non-malarial febrile illness as well. The objective of these studies was to assess acceptability of such a new pre-referral strategy by healthcare providers and likely uptake by the population.

**Methods:**

Two qualitative studies were conducted between May and July 2009. Study 1 investigated the acceptability of introducing a combined antimalarial-antibacterial suppository by interviewing 27 representatives of the three administrative levels (central government, regional, local) of the health sector; Study 2 investigated treatment-seeking behaviour and acceptability of this intervention at community level by interviewing 74 mothers in 2 villages.

**Results and Conclusions:**

Up to 92% of health representatives were in favour of introducing a new pre-referral strategy to tackle both malaria and non-malaria related severe illnesses in Guinea-Bissau, provided it was endorsed by international health authorities. The main obstacles to implementation were the very limited human and financial resources. In the two villages surveyed, 44% of the mothers associated severe illness with fever only, or fever plus one additional symptom. Mothers’ judgement of severity and ensuing decisions were not specific for serious illness, indicating that initial training to recognize signs of severe disease and treatment availability for non-severe, fever-associated symptoms will be required to prevent overuse of a new intervention designed as a pre-referral treatment for severely ill children. Level C health centres were the first resort in both villages (50% and 87% of respondents respectively). This information is encouraging for the implementation of a pre-referral treatment.

## Background

It has been estimated that *Plasmodium falciparum* malaria and acute respiratory infections (ARI) together account for 25% of childhood deaths worldwide. The clinical manifestations of malaria and ARI are often similar, which tends to lead to frequent overdiagnosis of malaria [1,2]. The health of a febrile child can deteriorate very rapidly and a large number of the deaths in rural areas occur before patients have reached a health care facility. In Tanzania, up to 40% of children who die have never been seen in a health centre; in Bolivia this figure approaches 74% [3]. In severe falciparum malaria, the risk of death increases in line with the delay in administering an effective anti-malarial drug and is maximal in the first 24 hours. Therefore, the speed at which appropriate management is instituted is the key factor influencing the outcome of treatment. Administration of rectal artesunate as a pre-referral treatment was evaluated in a large randomized, placebo-controlled trial of more than 12,000 malaria patients conducted in Bangladesh, Ghana and Tanzania. Such treatment reduced death or permanent disability in patients with severe malaria who experienced delays greater than six hours in reaching a health care facility [4]. Pre-referral treatment with a single dose of rectal artesunate has been introduced within the Integrated Management of Childhood Illnesses (IMCI) in remote areas.

Knowing that a sizeable proportion of deaths in young children labelled as malaria are probably the result of bacterial sepsis, in particular pneumonia, there is a strong argument for extending the strategy of emergency pre-referral treatment to antibacterial as well as anti-malarial therapy by co-formulating an antibiotic with an artemisinin derivative in suppository form [5,6]. Such a product has the potential to reduce mortality by reducing the delays in antibiotic treatment among children who can no longer take oral antibiotics.

Effectiveness of this strategy under real-life conditions not only depends on demonstrating efficacy of the formulation but ensuring access to treatment among remote populations who are likely to benefit most from this kind of intervention is a major challenge. This process starts with the incorporation of the new treatment into a country’s policy. Thereafter, a continuous supply of the drug in areas of need, should be established. In the rural tropics the nearest health centre is often several miles away from a village, thus the speed of detecting serious illness and patterns of treatment-seeking behavior that emphasize treatment and referral will also be factors affecting how a policy performs in practice.

The main objectives of this case study, which took place between May and July 2009, were: (i) to evaluate the acceptability of a proposed strategy of rectal antimalarial-antibacterial pre-referral treatment at all levels of the health sector; (ii) to evaluate the knowledge, attitudes and practices of mothers and primary health care staff confronted with febrile illness in children in rural areas in order to define implementation of home-based pre-referral treatment.

Guinea-Bissau was chosen because it has a high malaria prevalence and limited access to health structures for the majority of the population who live in rural areas (64%) [7]. The average life expectancy is 46 years and Guinea-Bissau ranked 164 out of 169 countries in the 2010 Human Development Index [8].

Infectious diseases account for most health care consultations, in particular malaria, ARI, diarrhoeal disease, HIV and tuberculosis. The first three of these account for 65% of deaths in children less than five years of age. More than 50% of medical consultations are for malaria, and the country reported 50 deaths for 100 000 admissions to health care facilities in 2012. Artemether – lumefantrine is the recommended treatment for uncomplicated malaria. Intra-venous quinine is the recommended treatment for severe malaria. ARI represents the second most common cause of death in children aged less than five years in Guinea-Bissau [9].

## Methods

Two qualitative studies were performed:

### Study 1 – Interviews with health sector representatives

#### Public health structure

The organization of the national public health service is pyramid-shaped with three levels, local, regional and central. The central level is represented by the Ministry of Public Health (MPH) and consists of the Office of Services, the National Programmes, Simão Mendes National Hospital and other national specialized hospitals and centres. The central level draws up policies and strategies, regulates health care activities and coordinates external aid. The regional level is represented by the Regional Hospital, Regional Health Office and regional health care teams. It is in charge of implementing the annual programme. At the local level, the health structures provide primary care and consist of Levels A, B or C depending on geographical location, access to a regional hospital, and size of population served:

• A: may serve also as secondary level of care, physicians, nurses, clinical, surgery and some laboratory diagnosis, referral system for the regional hospitals;

• B: secondary level of care, physicians, nurses, clinical and basic laboratory diagnosis;

• C: primary level of care, nurses only, first contact between community members and other levels of health facility. Very basic clinical care and no laboratory diagnosis.

These health care facilities are managed by a technical team and management committee. Representatives of the three levels of the health sector were interviewed (Table 1). The total number of respondents was 27.

**Table 1:** Description of interviewees in Study 1

**Category of respondent**	**Organization/Department**	**Position**	**Number**
Representatives of the Health Departments at central/government level	Department of Family Health	Director	1
	National Malaria Control Programme	Director	1
	National Programme For Health Development	Director of national control programme for malaria, HIV and tuberculosis	1
	National Programme For Health Development	Responsible for cost recovery	1
	Department of Primary Health Care	Director	1
	Department of Pharmacy and Medicines	Assistant Director	1
	CECOME*	Director	1
Representatives of the Health Departments at regional level	Regional departments of Health Oio Region	Director and Assistant Director	2
	CECOME* regional depot/distribution centre, Mansoa	Manager	1
Representatives of International organizations and donors	WHO Guinea-Bissau	Head of Department of Communicable Disease Control	1
	WHO Guinea-Bissau	WHO representative in Guinea-Bissau	1
	Médecins Du Monde	Medical coordinators	2
	UNFPA Guinea-Bissau	Director of Department of Reproductive health	1
	UNICEF Guinea-Bissau	Programme Director	1
	National Programme For Health Development	Global Fund representative	1
	Plan International	Medical coordinator	1
	WHO Guinea-Bissau	Malaria programme director	1
Health professionals at different levels	Simao Mendes National Hospital	Paediatrician	1
	Mansoa Regional Hospital	Paediatrician	1
	Farim Health Centre (Type A)**	Nurse	1
	Bissora Health Centre (Type B)**	Nurse	1
	Health Centre (Type C)**	Nurses	4

*CECOME Central de Compra de Medicamentos (Central Office for Purchasing of Essential Medicines, Guinea-Bissau).

**Health centre Type A, B or C: classification of primary health care centre according to activity. Level A has the most complete package of care including some surgery.

### Study 2- Interviews with mothers, community health workers, and health professionals from level C and B health care facilities (in rural Guinea-Bissau)

#### Selection of the community study sites and randomization

Tchale and Bantandjan villages in Oio region were selected. They were two of the furthest villages from a health centre with laboratory diagnostic facilities and ability to administer injectable treatment. The target population of this survey was mothers of children under five years of age. Inclusion criteria were availability during the study and informed consent of respondents. The sample size was 40 mothers per village.

Tchale village has around 1,000 inhabitants, mainly animists of the Balante ethnic group, and is situated 90 minutes (by foot) from a Level C health centre and three hours (by foot + car) from Bissora sector Level B health centre. The nearest health care facility able to manage severe malaria or ARI is Bissau National Hospital, three hours away from the village by road.

Each village consists of 32 plots distinct from each other; the number of houses per plot may vary from two to 21. At least one woman per plot and two women in larger plots were interviewed in order to reach a target of 40 respondents. Most women interviewed had never attended school. Apart from picking cashew nuts during the harvest season, most had no income-generating activities. The survey was conducted during the cashew nut harvest so many women were not at home. Thirty six housewives were interviewed in Tchale village, on the assumption that field- and home-working mothers shared responsibilities and income from each plot equally.

Bantandjan village is seven hours by foot or 75 minutes by car from a Level C health centre. The nearest reference structure is Mansoa Regional Hospital, situated between two hours and 15 minutes and eight hours from the village, depending on the type of transport used. Bantandjan has about 350 inhabitants who are mainly Muslims from different ethnic groups: Mandigoes, Manjaques, Mansanques, Pepels, and Balantes. The village is made up of 52 houses. One woman was interviewed in each of 40 households selected at random from a list of all village homes. Most women interviewed had never attended school. Apart from the palm oil harvest, they had no income-generating activities. The survey took place during palm oil season and 38 mothers were interviewed in Bantandjan village.

In each health area, but not in each village, there is community health services called Basic Health Structures (BHS), managed by Community Health Workers with very basic health training (no health related diploma), and traditional midwifes. The health care facilities selected for the study were close to Tchale and Bantandjan: four BHS, one Level C and one level B health centres surveyed and named by the mothers. The respondents of the health centre survey were health professionals in charge of these facilities. They were Community health Workers in BHS, nurses in in level C, and one doctor in level B health centres.

### Interview methodology

For both studies, interviews were semi-structured and conducted with the help of pre-prepared interview guides. The language of the interviews was French at the central level and Portuguese Creole at the regional and local levels, via translators. For study 1, open questions were used to evaluate acceptability of a pre-referral treatment at all health care levels. For study 2, open questions were used to evaluate the knowledge of the respondents of severely ill febrile children, and closed questions were used to define their attitudes and practices for treatment. The data collection tools were pre-tested in Bissau rural areas.

### Data analysis

Interviews were transcribed verbatim. Data collected was categorized by theme. Specific comments were selected either because they were repeated frequently or because they best reflected the overall opinion of the respondents on a specific topic.

### Ethical approval

The study protocol was approved by the Ethical Committee of the Department of Hygiene and Epidemiology of the Ministry of Health in Guinea-Bissau. Study participants were given a document outlining the background and aims of the project. Verbal consent was obtained before each interview. For interviews that were tape-recorded, permission was requested at the start of the interview.

## Results

### Study 1

#### Acceptability of the proposed strategy of a combined antimalarial-antibacterial emergency pre-referral treatment

All respondents judged that the proposed strategy was suitable for the epidemiological context in Guinea-Bissau for children less than five years of age. Several interviewees stressed their interest in having a combined medicine capable of treating both malaria and respiratory infections as a strategy to combat high childhood mortality rates. Some respondents remarked upon the pertinence of proposing a treatment which contains medicines already well known and conforming to the treatment protocols in place in the country, and in a form more suitable for young children than tablets. According to one paediatrician, the idea of a co-formulated treatment was particularly suited to Guinea-Bissau since there was already a national move towards integrated management of sick children for both malaria and respiratory infections in malaria-endemic regions. The current national health policy for rural areas has been trying to revive and strengthen the most basic peripheral health posts in an attempt to bring emergency treatment to the village level. Several respondents stressed the difficulties of geographical access that delay the arrival of patients at health care facilities, particularly during the rainy season. According to one respondent, a pre-referral treatment at community level might also help to counteract the delay in seeking treatment related to cultural practices:

‘At community level there are cultural difficulties because it is up to the father to take the decision to bring the child to a health centre. The mother waits for her husband to decide; that can take some time. This situation would justify the use of a pre-referral treatment in the community.’

The interviewees were aware that such a strategy would comply with WHO recommendations to use rectal artesunate as emergency treatment.

For 23 out of 25 respondents (92%) administration of a combined antibiotic-anti-malarial treatment would be an acceptable solution for the emergency pre-referral treatment of children aged less than five years. One single reservation mentioned was the risk that the advice to seek further treatment at a health centre would not be followed by parents once the suppository had been administered.

Two respondents remarked upon the acceptability of the strategy from a public health point of view in a context where there was no alternative. One of the paediatricians felt that a combination treatment containing an antibiotic would be an advance compared to giving artesunate alone. In practice, certain medicines are already given by the rectal route (quinine, phenobarbitone, paracetamol) without problems. The availability of a heat-stable suppository to treat children was considered advantageous. Some health professionals underlined the importance of giving clear information to mothers and community health workers on the use of this route of administration in order for it to be adopted easily.

#### Conditions for use of a combined antimalarial-antibacterial suppository formulation

According to the representatives of the various health-related institutions interviewed (six respondents), the introduction of such a treatment into the country would necessitate a modification of the policy for the management of malaria in children. This would imply an application to include the new medicine into the National List of Essential Medicines and the treatment policy documents of the National Malaria Control Programme and Integrated Management of Childhood Illness. This would be conditional on the identification of partners with the necessary financial and technical expertise.

The interviewees also discussed factors affecting access to treatment by the target population. Three main aspects were highlighted:

• For a large majority of the respondents, cost was the major determinant of accessibility to medicines. They referred to the strategy implemented to make Coartem® affordable to the population:

‘*The cost of the new medicine must not be higher than the cost of Coartem® ..(…) We know how much the population is able to pay and not all of them can afford Coartem®. We cannot give the medicine free of charge but the price must not be an obstacle.’*

• Half of the participants interviewed believed that a subsidy of a new medicine would be necessary in order to ensure accessibility.

• Half of those interviewed cited availability of the medicine as an important condition to ensure access to treatment. For them, the assurance of a regular supply from the manufacturer to the village was vital, with a reliable distribution system.

• Two of 6 respondents felt that access to the medicine should be guaranteed by the community health workers and basic health care units that are closest to the population.

In summary, the conditions necessary for the implementation of a new treatment in the health sector identified were:

• Demonstration of efficacy and/or heat stability (five respondents)

• Integration into the national treatment protocols (six respondents)

• Validation of the treatment by WHO (two respondents)

• Information, education and communication campaigns aimed at the population (18 respondents)

• Training, information and remuneration of the health technicians (18 respondents)

• Follow-up and supervision of the community health workers (five respondents)

• Population payment (token payment) towards the cost of the medicine (one respondent)

#### Objections, obstacles and difficulties anticipated for introduction in the country and for its use at community level

Interviewees postulated potential obstacles to the introduction of a medicine at the community level as follows:

• Poor availability of personnel at the community level: community health workers are already overworked and very often they do not stay a long time in the same village (four respondents)

• Financial difficulties of the population in rural areas which could prevent referral to health centres, because of transport, consultation and accommodation costs (three respondents)

• If the constituents of a new product were not already part of the national treatment policy for treatment of malaria and respiratory infections it would be difficult to get approval (three respondents)

• The administrative and financial burdens incurred when a new medicine is introduced into the national policy (two respondents)

• Cultural obstacles: reliance on traditional healers by the population in some areas (two respondents)

• The difficulties of ensuring a regular supply of a new medicine, particularly if the process involves external donors, as seen with the introduction of artemether – lumefantrine (Coartem®) financed by the Global Fund (one respondent)

• Problems of access to treatment in villages where there is no community health worker (one respondent)

• The contradiction between emergency treatment proposed with the planned introduction of rapid diagnosis tests for malaria in the peripheral health posts (one respondent).

### Study 2

#### Knowledge, attitudes and practices of mothers faced with a seriously ill febrile child Tchale village (36 respondents)

##### Knowledge of mothers regarding serious conditions in children

When asked to describe the signs and symptoms of a seriously ill child who may be dying, out of 36 mothers, 22.2% quoted fever only and 36% quoted fever plus one or more other symptoms Table 2).

**Table 2:** Signs and symptoms of serious illness reported by mothers - Study 2

**Symptoms quoted by mothers**	**% Tchale (n = 36)**	**% Bantandjan (n = 38)**
Fever only	22.2 (8)	7.9 (3)
Fever plus another symptom	22.2 (8)	36.8 (14)
Fever plus two other symptoms	8.3 (3)	18.4 (7)
Fever plus three other symptoms	5.5 (2)	2.6 (1)
Other symptoms – without fever	30.6 (11)	15.9 (6)
Don’t know	11.2 (4)	18.4 (7)
Total	100 (36)	100 (38)

Other symptoms: fatigue, vomiting, diarrhoea, anorexia, respiratory distress, prostration, convulsion, headache, icterus, pale face.

#### Attitudes and practices of mothers regarding serious illness in children

##### Treatment of first resort

When presented with a hypothetical situation in which their child is in a serious condition (febrile and cannot swallow) and asked to describe their first care-seeking response, 50% of 36 mothers said they would go to the Level C health centre and 27.8% answered that they would consult a traditional practitioner first (Table 3).

**Table 3:** Treatment of first resort in Tchale and Bantandjan

**First healthcare choice**	**% Tchale**	**% Bantandjan**
	**(n = 36)**	**(n = 38)**
Home treatment	5.6	2.7
Traditional practitioner	27.8	10.5
Level C health centre	50.0	86.8
Level B health centre	11.0	0
National Hospital	5.6	0

#### Various treatment-seeking behaviours described by the mothers for the treatment of their child and justification

**Figure 1:**
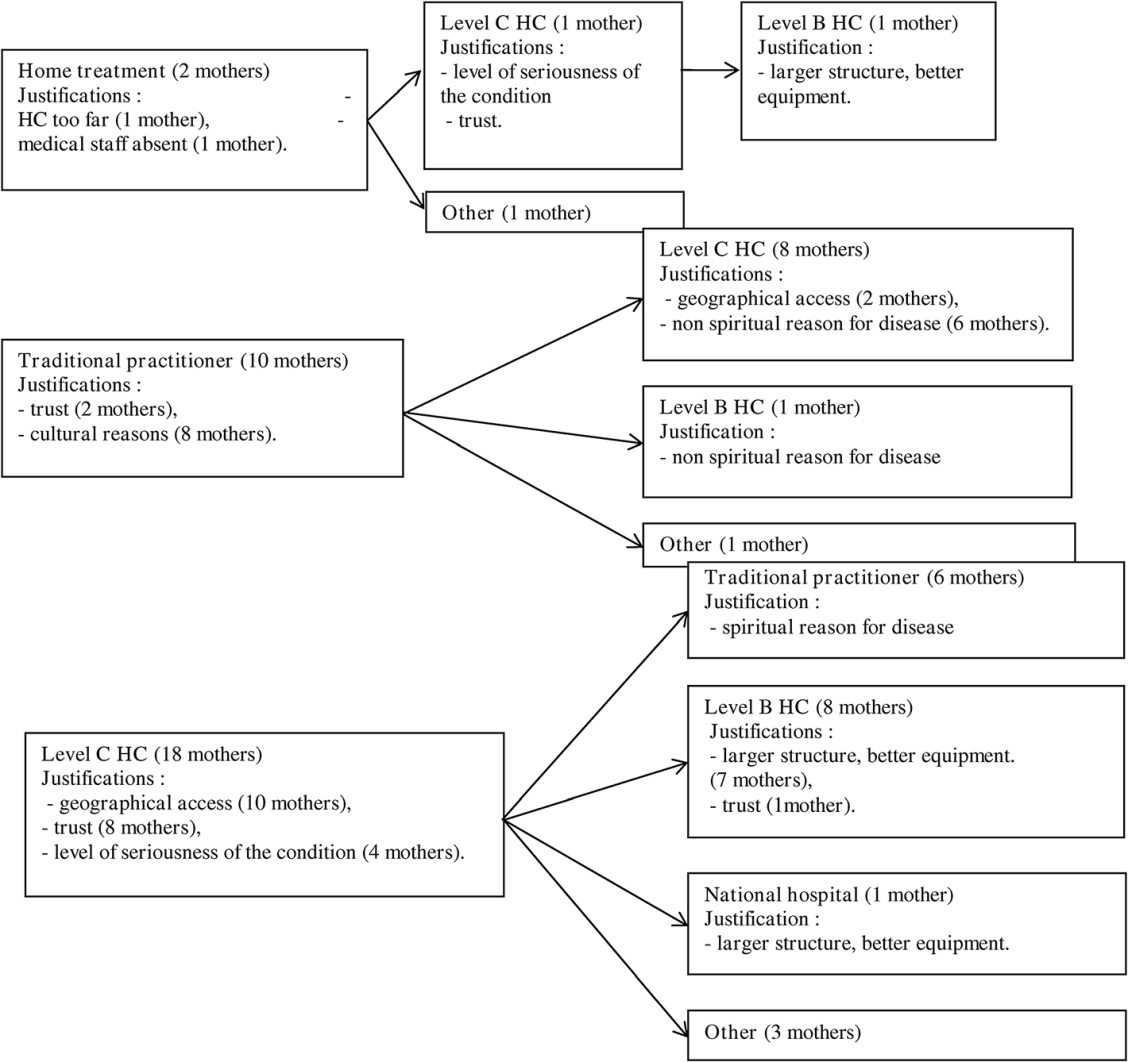
Patterns of treatment-seeking behaviour described by the mothers in Tchale.

When asked what they would do if their child was not cured after the first care-seeking response, most of the women who had opted for the traditional practitioner chose to go to a Level B or C health centre (Figure 1 and Table 3). For these women, the fact that the treatment provided by the traditional practitioner did not cure the child meant that the disease was not of spiritual origin.

Out of the 22 women who opted for a Level B or C health centre for the initial treatment, 31.8% said they would choose to consult a traditional healer next, while 50% would go to a higher level facility if the child had not improved after being treated in a Level C health centre.

#### Time spent in the village before departing for a health structure and justification

The delay before departing for a health facility varied from zero to seven days. The average lapse of time was 2.13 days (standard deviation = 1.58). Reasons given for the time delays were:

• To find money for consultation fees and drugs (83% of respondents)

• To find money for transport (61% of respondents)

• To find a means of transport (25% of respondents)

• To wait for the family decision-maker (2% of respondents)

• To be available to go (2% of respondents)

• Other (2% of respondents)

Two women stated they would not encounter any problems and could leave the village whenever they wanted to. Four mothers mentioned the need for a spiritual ceremony as one of the reasons for a delayed departure to a health facility.

#### Bantandjan village (38 respondents)

##### Knowledge of mothers regarding serious conditions in children

Of 38 mothers, 7.9% quoted fever only and 57.8% quoted fever plus one or more other symptoms. All mothers considered that a child unable to eat or drink was in a severe state (Table 3).

#### Attitudes and practices of mothers regarding serious conditions in children

##### Selection of the treatment of first resort

Out of 38 mothers, 86.8% said they would go to the Level C health centre and 10.5% answered that they would consult a traditional practitioner as a treatment of first resort (Table 3).

#### Various treatment-seeking behaviours described by the mothers for the treatment of their child and justification

**Figure 2:**
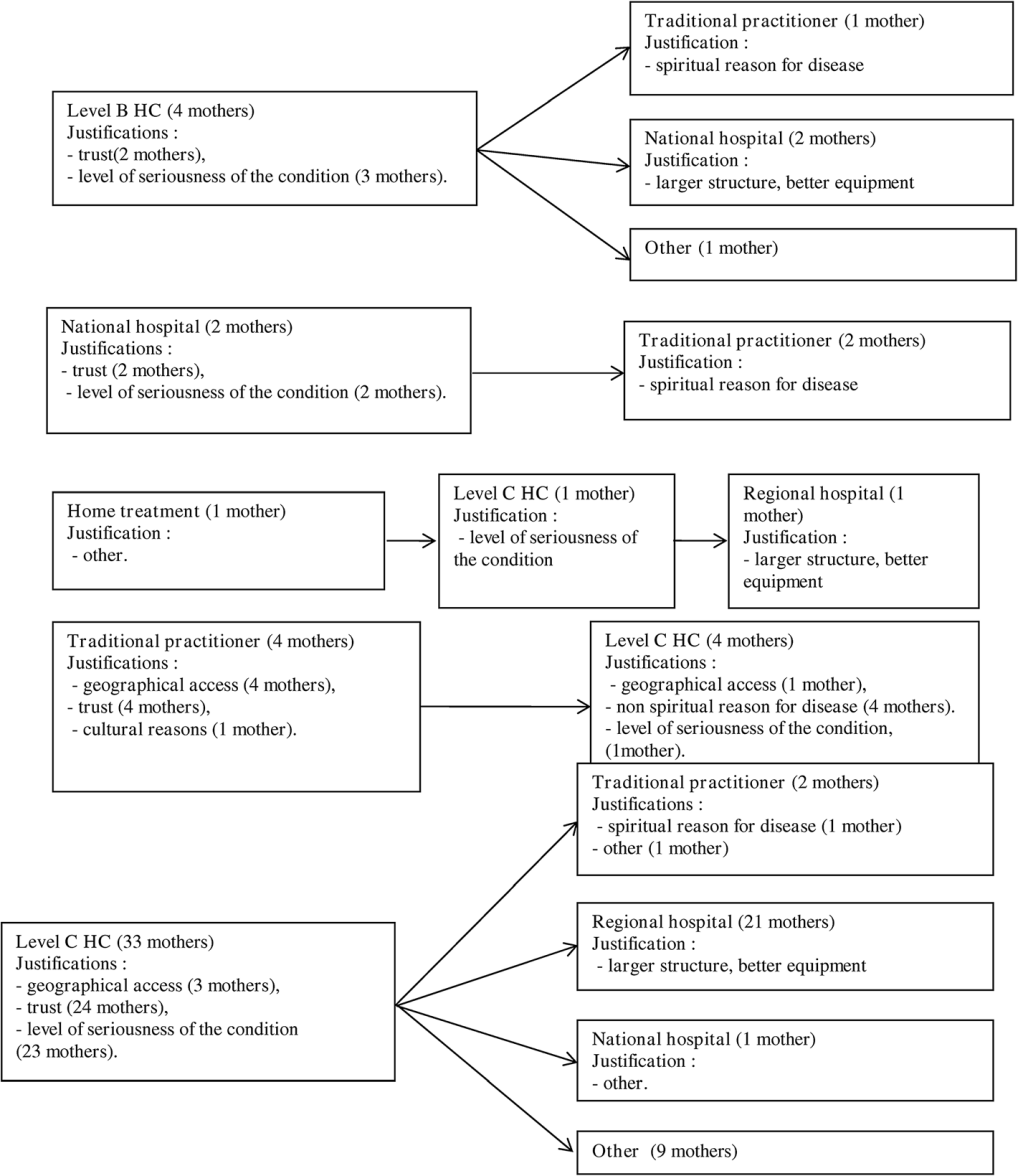
Treatment-seeking behaviour described by the mothers in Bantandjan.

The various itineraries described by the mothers with explanation are presented in Figure 2 and Table 3. The four women (11%) who opted for the traditional healer as a first resort would go to a Level C health structure if the child had not been cured by traditional ceremony. Of the 87% of women who opted for a Level C health centre as treatment of first resort, 6% would choose to consult a traditional practitioner and 67% would go to the regional or national hospital to have their child treated.

#### Time spent in the village before departing for a health facility and justification

The time delay in the village before departing for a health facility was shorter than in Tchale and ranged from zero to four days. The average lapse of time reported was 1.34 days (SD = 0.91). Reasons given for the delay were:

• To find money for consultation fees and drugs (97% of respondents)

• To find money for transport (97 of respondents)

• To find a means of transport (97 of respondents)

Only one respondent asserted that she would not encounter any problem.

#### Knowledge, attitudes and practice of staff of primary healthcare facilities managing complicated febrile illness in children

In order to provide complementary information, BHS, level C and one level B health care facilities were visited. In each of them, the person in charge was asked about his or her knowledge of serious conditions in children.

#### Features of a serious condition

The staffs in charge of health facilities were asked to describe the characteristic symptoms of a serious condition in children, apart from high fever. The respondents of the Level C health centres quoted convulsions and two of them also mentioned loss of consciousness. One doctor mentioned lethargy, facial oedema, vomiting, diarrhoea, loss of appetite, and weight loss. The community health officer interviewed from the BHS mentioned coughing, vomiting and diarrhoea.

#### Differential diagnosis for malaria and acute respiratory infections

BHS and level C health facilities did not have the capacity to carry out laboratory diagnosis and the community health officer had no knowledge of malaria rapid diagnostic tests (RDTs). Only one health centre had the necessary equipment to perform a blood smear. The diagnosis of ARI or malaria was based on clinical assessment in the three health facilities visited. In BHS, out of four health professionals interviewed, two did not know about RDTs. Indeed, RDTs were not part of the list of medical equipment provided to BHS or first level C health centres. Each health officer had a different description of ARI and malaria symptomatology, although in most cases fever was associated with malaria while cough and abnormal respiratory rate were associated with ARI.

#### Description of health care provision

When a febrile child who cannot swallow consults a BHS, the community health officer systematically refers him to the nearest health centre. No treatment is administered to the child before referral. In the Level C health centres unable to perform laboratory diagnosis, basic resuscitation is provided and a child is then sent to the nearest reference health centre. At one of those centres the only pre-referral treatment received by a child is diazepam given by intramuscular (IM) injection in case of convulsions. The nurse then refers the child, which in most cases means the mothers have to walk carrying their child to the referral facility. In the other two centres, the treatment of a febrile child unable to swallow usually consists of a quinine drip with 5% glucose. In one of those centres, the staff will also administer paracetamol by IM injection or as an addition to the parenteral solution. Referral is immediate, or within an hour of receiving intravenous (IV) therapy depending on the centre. In health centres managed by a doctor, the child is treated on site. On arrival, the child receives symptomatic presumptive care, while waiting for the result of blood smear test. The treatment consists of IM injections of paracetamol and/or diazepam in case of convulsions. If the blood smear is positive, the child is treated according to the treatment protocol for severe malaria, i e, IV quinine with 5% glucose. If the blood smear test is negative, the doctor tries to diagnose alternative pathologies depending upon the clinical presentation. If the child is assessed as having acute respiratory infection, treatment is normally ampicillin IV injection for seven days. The child is put under observation in the health centre for two days. If there is no improvement after two days, the family is referred to Simao Mendes National Hospital in Bissau.

## Discussion

The proposed strategy of introducing a combined antimicrobial-anti-malarial emergency pre-referral treatment was acceptable by health sector representatives and was felt to be well adapted to the context in Guinea-Bissau. This approach was also considered to fit well with the current move to revitalize the most peripheral health facilities and would be in line with WHO recommendations for the use of pre-referral treatment with rectal artesunate [10]. Use of the rectal route to deliver medicines was not perceived to present a problem and in fact this approach was considered to fill a gap since, currently, there is no emergency treatment option available for sick febrile children in remote areas of Guinea-Bissau.

In studying the knowledge, attitudes and practices of mothers in Tchale and Bantandjan villages and health professionals in the nearest primary health care facilities, the objective was to describe the real conditions in which any future pre-referral rectal treatment would be used. The questionnaire did not intend to investigate the perception of intra-rectal drug administration at the village level. Nevertheless, informal discussion with the health representatives indicated that rectal administration of drugs was not a problem in the country. Similar compliance to rectal administration has been described in other West African countries [11,12]. However, a study performed in Laos indicated reluctance by the Lao community to use the rectal route, thus highlighting the need to take in account local behaviour before introducing a new therapy [13].

In Guinea-Bissau, all mothers acknowledged that children who could not swallow were in a serious condition. On the other hand, some mothers described such a variety of signs of illnesses that there would be a real risk that an emergency treatment might be too late and thus not guarantee a child’s survival during transportation to the reference health centre if its use was restricted to very severe cases. If a new treatment does not prove its efficacy, there is a possibility it might not be accepted by the community.

As for the treatment-seeking behaviour, 66.7% of the mothers interviewed in Tchale and 86.8% of those interviewed in Bantandjan declared they would go to a health structure when their child was in a serious condition. These women were not opposed to a pre-referral emergency drug treatment readily for their child. However, 27.8% of the mothers interviewed in Tchale and 10.5% of those interviewed in Bantandjan said they would first opt to consult a traditional healer. Tchale mothers selected this option for cultural reasons despite the proximity of a level C health centre, compared to Bantandjan. It is possible that the mothers with strong spiritual beliefs would be more reluctant to accept the administration of the drug as the treatment of first resort. Indeed, a study conducted in Tanzania on rectal artesunate has shown that the perception of the causes of the diseases might influence the implementation of an early treatment [14]. Bantandjan women chose to consult a traditional healer mainly because there was no level C health facility in the village and access to the first health centre was difficult for geographical or financial reasons. It is anticipated that these women would not be so reluctant to consult a community health worker in charge of dispensing a life-saving drug in the village.

Most of the Tchale mothers and all of the Bantandjan mothers making the trip to the health centre chose a Level C health centre. A survey in these facilities showed that, in most cases, the Level C health centre does not have the capacity to perform laboratory diagnosis, or to initiate appropriate drug treatment. Furthermore, most of these centres refer seriously ill children to the next facility after dispensing emergency first aid.

We did not probe the fact that, by definition, pre-referral treatment would need to be followed by referral to a health facility in our study. Yet, provision of rectal artesunate or a combination-therapy that treats both malaria and ARI with emergency pre-referral combination therapy treatment, means that a suppository dispenser in the village would have to direct the mothers towards a Level A or B health centre or the hospital. Consequently, mothers of treated children would need to accept the need for referral and agree to facilitate transit of the child to referral facilities which are located further from the village than basic health units. A study of the factors influencing compliance with referral advice after pre-referral treatment in Tanzania has shown that if the child presents signs of serious illness such as coma or convulsions, transit to a referral health facility was increased three-fold [15]. Our results here show that there can be a substantial time delay before departing to a health facility (average of 2.13 days in Tchale and 1.34 days in Bantandjan) because of poor transport and lack of money. Even if the treatment was available in the village, mothers of sick children would still need to comply with referral advice- and mechanisms for facilitating referral were not explored in our study.

## Conclusion

Guinea-Bissau has the infrastructure necessary to make a new medicine available at community level. The conditions for introducing a new treatment were clearly laid down by study respondents and related to properties of the medicine itself (efficacy, heat stability, cost, availability, validation by health authorities) and its integration into the health system (revitalization and expansion of the network of health care providers at community level, availability and training of health care workers). The ability of mothers to recognize early signs of severe illness is a key factor favouring use of pre-referral treatment. On the other hand, this would imply providing, in parallel, oral artemisinin-combination treatment for mild malaria to reduce the evolution of disease to severe malaria, and early diagnosis and treatment of pneumonia via oral antibiotics to prevent evolution to severe pneumonia.

## Competing interests

The authors declare that they have no competing interests.

## Authors’ contributions

AV passed away on December 3, 2013. This article was seen and approved by her for initial submission to the Malaria Journal. AV and AL carried out the study in Guinea-Bissau; AR coordinated the study in Guinea-Bissau; NJW, EAA, MG, PO, and PM conceived the study, participated to its design and coordination, and helped draft the manuscript. All authors read and approved the final manuscript.
